# Medin, a link between vascular pathology and dementia?

**DOI:** 10.1177/0271678X241289772

**Published:** 2024-10-07

**Authors:** Ilse S Altenburg, Nina G Smets, Gustav J Strijkers, Erik NTP Bakker

**Affiliations:** 1 Department of Biomedical Engineering and Physics, Amsterdam University Medical Center, Amsterdam, The Netherlands; 2 Amsterdam Cardiovascular Sciences Research Institute, Amsterdam, The Netherlands; 3 Amsterdam Neuroscience Research Institute, Amsterdam, The Netherlands

**Keywords:** Amyloid β deposition, CAA, dementia, medin, vascular pathology

## Abstract

Medin is a protein fragment derived from milk fat globule epidermal growth factor VIII (MFG-E8). Medin aggregates are present in the vessel wall of most subjects over 50 years of age. In this narrative review, we focus on the consequences of medin aggregation in relation to the development of dementia. Recent literature revealed medin as biomarker for dementia in CSF, specifically of a vascular subtype. Preclinical work showed that medin is associated with aging-related cerebral vascular dysfunction, vascular stiffening, hypertension, and. vascular amyloid β deposition. These findings position medin as a potential mechanistic link between aging, vascular pathology and dementia.

## Introduction

Vascular cognitive impairment frequently coexists with neurodegenerative diseases, such as Alzheimer’s disease (AD).^1^ Vascular cognitive impairment may result from both large- (i.e. arterial occlusions) and small vessel disease, such as microvascular dysfunction, micro-infarcts and microbleeds.^2,3^ Cerebral amyloid angiopathy (CAA) is defined by vascular amyloid β deposition.^4^ CAA can affect both large and small vessels, and forms an important contributor to vascular cognitive impairment.^3^ Aging, but also hypertension, are recognized as risk factors for cerebral vascular pathology.^5–7^ However, the precise mechanisms through which these risk factors affect the vasculature and ultimately lead to dementia remain largely unexplored. In this concise narrative review, our objective is to summarize recent research advancements on medin, focusing on its identification as a biomarker for various types of dementia and its involvement in cerebral vascular pathology. We will argue that medin may provide a mechanistic link between aging, vascular pathology and dementia. Furthermore, we identify current gaps in knowledge regarding medin, providing directions for future research.

### Medin, a protein fragment cleaved from milk fat globule epidermal growth factor VIII

Milk fat globule epidermal growth factor VIII (MFG-E8) is a protein that is part of the milk fat globule membrane. MFG-E8 is however not only present in milk, but also in other tissues and body fluids, including blood and cerebrospinal fluid. This is the result of its expression by various cell types such as vascular smooth muscle cells (VSMCs), macrophages, microglia and astrocytes.^8–11^ MFG-E8 is a 378-amino acid glycoprotein consisting of three characteristic domains. First, an epidermal growth factor (EGF)-like domain located at the N-terminus, followed by two domains with strong sequence homology with the C1 and C2 domains in the coagulation factors V and VIII.^12^ Medin is located within the C2-like domain^13^ and cleaved from the full protein, through mechanisms that are yet to be identified. MFG-E8 has a number of biological functions, but its role in milk and the mammary gland will not be further discussed in this paper. Other important functions include its role in phagocytosis and in coagulation. Thus, the resemblance of parts of MFG-E8 with coagulation factors probably explains its anticoagulant properties, as it may act as competitive inhibitor for binding of these coagulation factors to membranes.^14^ The role of MFGE-8 in phagocytosis^15^ may also be relevant in the context of dementia.^16,17^ The focus of this review, however, will be geared towards the role of medin in the vascular contribution to dementia.

### Medin as novel biomarker in CSF

CSF and blood biomarkers aid in the diagnosis of dementia and its subtypes. As medin levels were found to be increased in arterioles of patients with either AD, vascular dementia, or a combination of both, as compared to healthy elderly, it was suggested as a novel biomarker.^18^ Recently, this suggestion was confirmed and refined in a study by Tijms et al.^19^ These researchers discovered five different subtypes of Alzheimer’s disease based on extensive CSF proteomics. One of these subtypes was associated with neuronal plasticity, reporting an increase in MFG-E8 levels in this subtype compared to healthy controls.^19^ Another subtype was defined by alterations in proteins related to blood-brain barrier (BBB) dysfunction. This subtype was characterized by the leakage of blood specific proteins such as albumin and IgGs in the cerebrospinal fluid, but showed reduced MFG-E8 levels, next to a reduction in other vascular proteins in CSF. Thus, it appears that MFG-E8 levels may either increase, decrease, or remain unaltered, depending on the underlying etiology leading to AD. MFG-E8 could therefore be a valuable biomarker in discriminating subtypes of AD.

While speculative, the ‘BBB subtype’ of AD in the study of Tijms et al.^19^ may reflect a subset of patients that suffer from concomitant CAA. Indeed, patients of this subtype showed a relatively high occurrence of microbleeds, a feature consistent with the presence of CAA. This hypothesis is consistent with data from a study by Marazuela et al.,^20^ where a distinction between AD and CAA patients was made based on the modified Boston criteria. Indeed, CAA patients had significantly lower CSF levels of MFG-E8 compared to AD patients and healthy controls. Upon histological evaluation, strong staining for MFG-E8 was found in amyloid β positive vessels from CAA patients. The same study reported similar findings in a mouse model. Thus, APP23 mice showed significantly higher MFG-E8 levels when aging compared to the age-matched wild type mice, while also showing more MFG-E8-positive blood vessels in APP23 mice. Interestingly, MFG-E8 positive staining was only found in amyloid β positive vessels, and not in parenchymal amyloid β plaques. Taken together, these studies show that both human patients suffering from CAA and animal models of CAA show strong vascular staining of MFG-E8, while CSF levels are reduced. Possibly, this could be due to retention in the vessel wall, perhaps after cleavage and aggregation in the form of medin.

### Medin aggregates in arteries of aged humans

It is unclear what determines the cleavage of MFG-E8 and subsequent formation of medin aggregates, but evidence shows it is strongly age related. The group of Westermark led the way in identifying medin as an amyloid protein in human arteries, including cerebral vessels. Following the extraction of medial amyloid from a small group of older human subjects, the authors pinpointed medin as the primary component.^21^ In the same paper, they also showed that MFG-E8 was present in aortic medial cells using immunostaining, suggesting that vascular smooth muscle cells are the primary source. Finally, they showed that a synthetic peptide corresponding to the c-terminal part of the amyloid had a strong tendency to form fibrils in vitro. In a subsequent study in a much larger group of patients, Westermark and colleagues showed that medin was present in the aorta.^22^ The latter study included samples of thoracic artery derived from 18 patients, ranging from 57 to 88 years of age. Interestingly, the authors noted that medin aggregates were found in arteries of the upper body alone, such as the temporal, coronary and basilar arteries, in addition to the thoracic aorta. The association of MFG-E8 with age was further confirmed in a proteomic screening of aortic samples from young, middle-aged, and old subjects by Miura et al., showing a 7-fold increase in MFG-E8 expression in old vs. young subjects, paralleled by amyloidosis in the vessel wall.^23^

**Figure 1.**
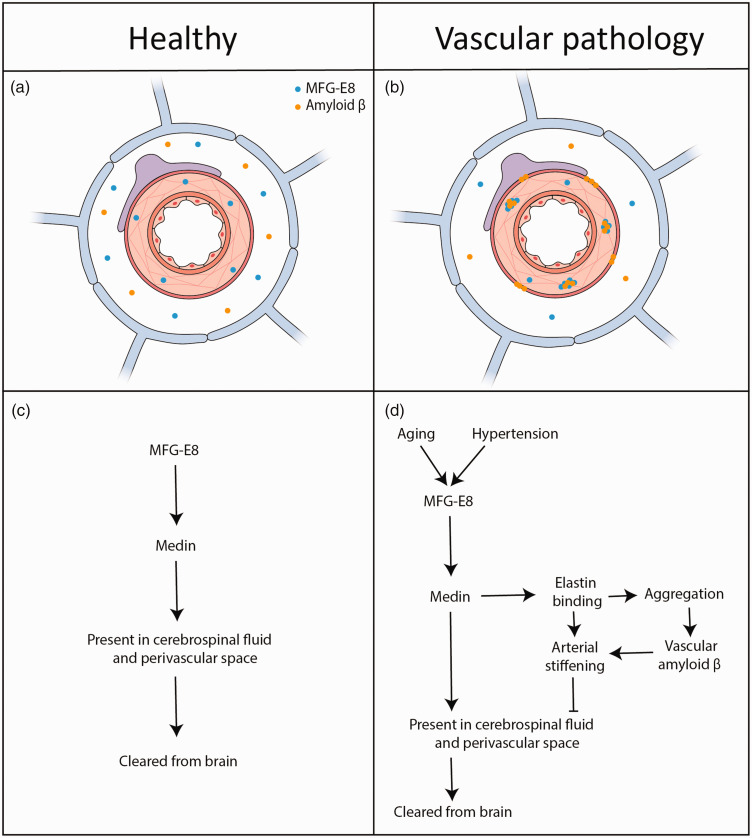
Simplified schematic overview of medin and amyloid ß interplay in relation to brain clearance under healthy and pathological conditions. (a) MFG-E8 and amyloid ß distribution in a healthy cerebral artery and perivascular space. Both proteins may be cleared from the brain via perivascular spaces to the CSF, offering the potential for detection and use as biomarker. (b) Under pathological conditions and upon aging, MFG-E8 may be deposited as medin aggregates in the vessel wall, which is associated with amyloid ß plaque formation. (c) Proposed medin clearance pathway in healthy tissue and (d) overview of the proposed sequence of events leading to medin deposition and vascular amyloid β aggregation in aging and hypertension.

In another study by Westermark et al., immunostaining demonstrated the association of both MFG-E8 and medin with elastin fibers in the human aorta.^13^ Binding of medin to tropoelastin, a precursor of elastin, was demonstrated in vitro in the same work. Such binding to elastin led to the suggestion that medin may cause arterial stiffening, a hallmark of aging. However, a study specifically addressing this question did not show a difference in MFG-E8 expression in segments of aorta with high vs. low pulse wave velocity, an indicator of vascular stiffness.^24^ Additional work is therefore needed to investigate the relationship between aging, medin deposition, and vascular stiffness in humans (Figure 1).

### Medin induces vascular dysfunction

As studies in humans concerning medin were mainly descriptive, evidence for its causal role in vascular pathophysiology was falling behind. Recently the group of Neher conducted studies in mice that addressed the role of medin. Using wild type and medin knockout mice, it was demonstrated that similar to humans, mice showed an increase in MFG-E8 expression and accumulated medin in both aorta and cerebral vessels upon aging.^25^ Interestingly, medin knockout mice were resistant to age-related vascular dysfunction. Wild type animals showed a decrease in functional hyperemia upon aging, which was prevented in knockout animals. The specific aspect of neurovascular coupling impacted by medin was unclear, since the integrity of endothelial cells, smooth muscle cells, and astrocyte coverage of the vessels was maintained. However, explanations for these findings might be found in the work of other groups. Specifically, evidence indicates that medin may cause a shift towards an inflammatory vascular phenotype,^26^ and impairs endothelium-dependent dilation through an altered balance in nitric oxide and superoxide production.^27^ Whether vascular stiffness is altered in medin knockout mice, remains to be established.

### Medin promotes vascular amyloid deposition

In a recent paper, Wagner et al. reported the important discovery that medin promotes vascular deposition of amyloid β.^28^ Their research demonstrated that eliminating medin in two mouse models resulted in decreased amyloid β deposition. Notably, this reduction was especially pronounced in the APP23 mouse model, where both amyloid β deposition and overall pathology significantly decreased. This model is characterized by vascular amyloid β deposition and therefore considered a model of CAA. Similar to this mouse model of CAA, the same authors showed that in human samples, MFG-E8 was associated with blood vessels and increased levels of the protein fragments were found in cases of Alzheimer’s disease with strong presence of CAA. Next, it was shown that medin aggregated together with amyloid β in vitro, and structural modeling confirmed a favorable interaction of the two peptides. Finally, seeding of medin aggregates derived from human aortic extracts, or from aged WT mice, into the brains of mice significantly increased amyloid β deposition several months later. Taken together, this work provided novel mechanistic insight in the role of medin, by promoting vascular deposition of amyloid β. This appears particularly relevant in the context of CAA. Some caution however is appropriate as to how medin leads to amyloid β deposition. The authors hypothesized that binding of medin to amyloid β is the main cause of amyloid β deposition, but the role of MFG-E8 in phagocytosis should also not be ignored. Although in the paper of Wagner et al. no effect on microglial phagocytotic function was found in MFG-E8 knockout animals,^28^ data suggest that MFG-E8 participates in uptake of amyloid β by macrophages and microglia.^29,30^ If this function is hampered in MFG-E8 knockout mice, it could provide an alternative explanation for amyloid β aggregation.

### Does hypertension increase MFG-E8 and medin?

Next to aging, hypertension is a strong risk factor for cerebrovascular disease and cognitive decline, amongst others via the development of arteriosclerosis. This raises the question whether hypertension also induces MFG-E8 expression and/or medin deposition. Data on this topic is scarce but affirmative. Proteomic screening of small mesenteric arteries from spontaneously hypertensive rats identified MFG-E8 as the most upregulated extracellular matrix protein.^31^ Similarly, in cerebral arteries MFG-E8 was found to be increased in hypertensive rats as compared to normotensive controls,^32^ which was related to an imbalance in angiogenesis in hypertensive vs. normotensive rats. However, a connection with vascular stiffness or dysfunction was not addressed. However, arteries of these hypertensive rats are known to show hypertrophic remodeling and reduced distensibility, with altered properties of elastin.^33^ These vascular changes fit the description of actions attributed to medin, but definitive proof of its role in vascular remodeling associated with hypertension is lacking. Additional circumstantial evidence for a role of MFG-E8 in hypertension comes from a study on cultured smooth muscle cells. Herein angiotensin II, part of the renin-angiotensin system, was found to stimulate MFG-E8 expression, resembling the effect of hypertension on blood vessels.^34^ Thus, given the limited research on the link between MFG-E8 and hypertension, these initial findings underscore the need for further investigation.

### Conclusions and future directions

Taken together, medin, a fragment of MFG-E8, is the most abundant amyloid protein in arteries of aging subjects known thus far. Recent studies exploring human cerebrospinal fluid identified MFG-E8 as a biomarker of dementias associated with vascular dysfunction. This includes CAA and a specific subtype of Alzheimer’s disease, characterized by blood-brain barrier dysfunction. It remains to be established whether this ‘BBB subtype’ actually reflects CAA. The results of biomarker studies align well with preclinical work that showed medin facilitates amyloid β deposition in the arterial wall. As MFG-E8 is expressed by vascular smooth muscle cells, this could also explain the preferential deposition of amyloid β in arteries as opposed to veins. Not only aging, but also hypertension may increase the expression of MFG-E8 in the arterial wall. Whether hypertension is associated with increased medin formation and aggregation in the vascular wall is currently unknown. It is also unclear which effects of medin on vascular structure and function are most important in dementia. Not only increased vascular deposition of amyloid β, but also vascular stiffening, and endothelial dysfunction may contribute. The role of medin on vascular stiffness should be better supported, as the evidence that medin affects this parameter seems mostly a derivative of its binding to elastin. An alternative mechanism, for instance, could be the binding and concerted action of medin with tissue-type transglutaminase.^30^ This enzyme was reported to induce vascular stiffening^35^ and facilitate protein aggregation in Alzheimer’s disease.^36^ Finally, vascular stiffness could affect clearance of amyloid β and other waste products along perivascular spaces, as depicted in Figure 1.^37^ The link between BBB dysfunction and MFG-E8 should also be further studied. Endothelial dysfunction caused by medin could affect the integrity of the blood-brain barrier, but data on this topic are lacking. Clinically, insight in how medin is cleaved from MFG-E8, and how MFG-E8 expression is regulated and can be manipulated,^38^ could be very beneficial for developing treatments of neurodegenerative diseases.
